# A New Approach to Explore the Surface Profile of Clay Soil Using White Light Interferometry

**DOI:** 10.3390/s20113009

**Published:** 2020-05-26

**Authors:** Suchun Yang, Junwei Liu, Longfei Xu, Mingyi Zhang, Dong-Sheng Jeng

**Affiliations:** 1 School of Civil Engineering, Qingdao University of Technology, Qingdao 266033, China; ysc116688668@gmail.com (S.Y.); zhangmingyi@qut.edu.cn (M.Z.); d.jeng@griffith.edu.au (D.-S.J.); 2 Key Laboratory of Special Area Highway Engineering, Ministry of Education, Chang’an University, Xi’an 710064, China; Longfei.xu@chd.edu.cn; 3 School of Engineering and Built Environment, Griffith University, Gold Coast, QLD 4222, Australia

**Keywords:** white light interferometry, clay soil, high compressive stress, porosity, contact region

## Abstract

In order to have a better understanding of the real contact area of granular materials, the white light interference method is applied to explore the real surface morphology of clay soils under high stress. Analysis of the surface profile indicates that there exists a support point height *z*_0_ with the highest distribution frequency. A concept of a real contact region (from *z*_0_ to *z*_0_ + *d*_90_; *d*_90_ represents the particle size corresponding to 90% of the volume fraction) is proposed by combining a surface profile with the particle size distribution of clay soil. It was found that under the compressive stress of 106 MPa–529 MPa, the actual contact area ratio of clay soil varies between 0.375 and 0.431. This demonstrates an increasing trend with the rise of stress. On the contrary, the apparent porosity decreases with an increasing stress, varying between 0.554 and 0.525. In addition, as the compressive stress increases, the cumulative frequency of apparent profile height (from *z*_0_ − *d*_90_ to *z*_0_ + *d*_90_) has a concentrated tendency with a limited value of 0.9.

## 1. Introduction

The contact characteristics of granular materials are important for prediction of friction between interfaces in geophysical, geological, petroleum, ocean and geotechnical engineering [1,2,3,4,5,6]. Clay particles are widely distributed in the mainland and ocean. In view of the interfacial friction of clay soil, the theory of “adhesion-ploughing” [7] can be used to explain the macroscopic friction force composition with meso-physical quantities. Friction is the resistance encountered when two mutually contacting surfaces are sliding or rolling. There are numerous factors affecting friction, and lots of researchers explain the phenomenon of friction by the early mechanical interlocking theory and molecular attraction theory within a certain range. However, interpreting friction as a single factor has a one-sided effect and does not work well for the hard-soft interface. Bowden and Tabor [7] proposed the adhesion-ploughing theory to lay the theoretical foundation for modern solid friction. Their theory is based on the joint action of mechanism and molecules. With the continuous improvement of the theory [8,9,10,11], it can be well applied to the hard-soft interface.

The study of tribological behavior of granular materials has been remarkably developed in recent years [12,13,14,15,16,17,18,19,20,21,22,23,24,25,26]. There have been theoretical advances, including discrete element method and modern tribology theory. The discrete element method can better simulate the application of granular materials, but the constitutive model relationship between granular materials needs to be further explored [27,28,29,30,31,32]. Interface friction has been studied for a long time and several modern tribology theories have been proposed simultaneously. These theories can well explain the composition of the interface friction formed by a continuum, but they still need further systematic work to better explain the composition of the interface friction formed by discrete particles.

On the other hand, the continuous improvement of meso-testing methods, such as SEM (Scanning Electron Microscope), TEM (Transmission Electron Microscope), AFM (Atomic Force Microscope), and other testing methods [33,34,35,36], can be used to conduct mesoscopic research on granular materials. Numerous scholars [36,37,38,39,40,41,42,43,44,45] applied advanced experimental methods to study granular materials and explore the friction characteristics of granular materials. It has been reported in the literature that the frictional force at the interface of granular materials is generated by ploughing [17], and the actual contact stress between particles is 0.8–2 Gpa. The magnitude of the ploughing force is closely related to the actual contact area. The friction at the interface depends on the number of contact points between the particles, that is, the total contact area of all contact points.

**Figure 1:**
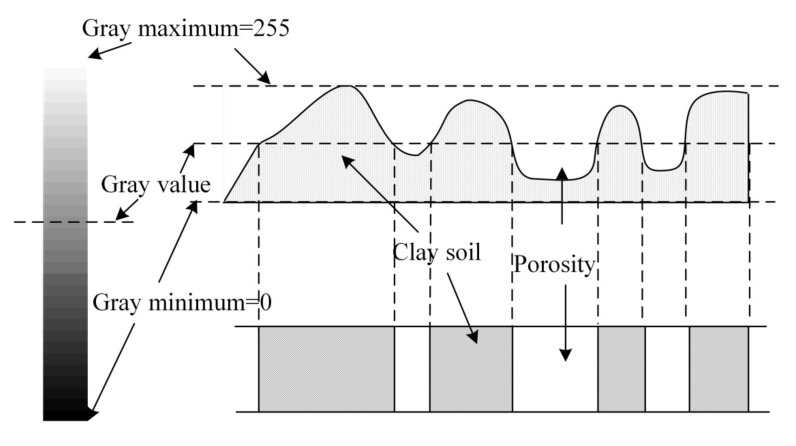
Principle diagram of the binarization method.

It is obvious that the actual contact area *A_r_* is indispensable in the constitution of macro-friction [46,47,48,49]. In order to explore the actual contact area of granular materials [50,51,52,53,54,55], many researchers have applied the binarization method to the gray image produced by scanning electron microscopy, thereby establishing a 3D model. The real contact area ratio (CAR), which is defined as the ratio between the real contact area and the total interface area, is finally quantitatively calculated. The specified principle of the binarization method is illustrated in Figure 1. However, the arbitrary choice of gray value will cause significant variations of contact area ratio.

For non-contact measurement methods of thixotropic samples, the original appearance of the samples can be better maintained. It is difficult for an optical microscope to form a clear image through the sample, and the white light interference technique [56] can scan the profile height information of the sample surface well. Especially in the medical field, it is applied to in vivo measurement [57], which reflects the non-damaging advantage of non-contact measurement. White light interference technology was proposed in the 1990s [58] and has been widely applied in the fields of machinery [59], medical treatment, chemistry [60], civil engineering [61], and precision manufacturing. However, there are few relevant references in the field of geotechnical engineering.

In order to accurately capture the real contact area, white light interferometry was introduced to explore the surface profile of the clay soil interface under different compressive pressures, using non-contact measurement with nano-precision. To the authors’ best knowledge, this is the first study of its kind with an aim to understand the microscopic changes in the surface contact area of clay soil.

The aim of this study is to explore the real source of interface resistance through the constituent elements. The present paper is organized into the following sections: in Section 2 and Section 3, the adhesion–ploughing friction theory will be outlined and the composition of the interface friction resistance will be discussed. In Section 4 and Section 5, the surface profile of the clay soil under normal load will be tested by the white light interference technique, which also explains real contact area.

## 2. Adhesion–Ploughing Friction Theory

### 2.1. Basic Concept

Under loading, the contact point of the two asperities undergoes plastic deformation due to excessive pressures, and the contact points are firmly adhered into one, which is called adhesion. When adhesion occurs, two surfaces of the asperities slide relatively, the adhesion point is sheared, and the shearing force is the adhesion resistance in the frictional resistance.

When the surface of asperities with different hardness contacts and relative sliding occurs, hard asperities will generate ploughing on the surface of soft asperities, and the ploughing resistance at the asperities is another part of the frictional resistance.

The source of frictional resistance [62] is divided into two parts based on the adhesion–ploughing friction theory, namely, adhesion resistance and ploughing resistance. The generation of adhesion resistance mainly depends on the number of contact areas, and the number of contact areas is highly related to the normal load and surface profile. The ploughing resistance mainly depends on the surface profile height of the soft and hard interface. In the final analysis, the height of the surface profile and the magnitude of the normal load are the dominant factors that determine the resistance.

### 2.2. Surface Interaction

#### 2.2.1. Adhesion Effects

**Figure 2:**
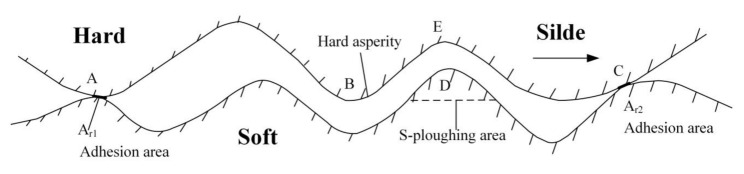
Adhesion and ploughing.

Since the actual contact area only occupies a small percent of the apparent contact area, the stress at the actual contact point reaches the yield strength *σ_s_* and plastic deformation occurs, and the actual contact point is firmly adhered by the compressive stress. Because the stress of the actual contact points has reached the yield strength, the loading increment can only be borne by increasing the actual contact area (as asperity A,C in Figure 2).

New adhesion points will be continuously formed during the relative sliding of two surfaces regardless of the adhesion points cutting. With the alternation of the old and new adhesion points, total adhesion area of the adhesion points will remain unchanged.

#### 2.2.2. Ploughing Effects

When these two asperities relatively slide, a variety of material migration phenomena may occur at the contact points due to the ploughing, which is related to the hardness of the asperities material (as ploughing asperities A,B in Figure 2). The material migration phenomenon is mainly divided into the following:

1. When asperity B (or A) passes through asperity D, asperity D undergoes relatively large plastic deformation and becomes adhesive. If the adhesive strength between the hard and soft medium is greater than the adhesive strength of the soft medium, sliding shear occurs inside the soft medium, making the soft medium transfer from the lower surface to the upper surface (asperity D is ploughed and adhered to the right side of asperity B, even filling asperity B).
2. When asperity B (or A) passes through asperity D, it undergoes plastic deformation. If the adhesive strength between the hard and soft medium is smaller than the adhesive strength of the soft medium, the adhesion between the soft and hard medium is not firm (asperity D is ploughed and cannot be adhered to B). Asperity B (or A) ploughs along D, which means cutting along the interface. At this time, material migration and deformation (ploughing) instead of transfer occurs at asperity D.
3. Asperity D only produces elastic deformation, and asperity B (or A) slides through D more easily than D produces plastic deformation.

### 2.3. Adhesion–Ploughing Calculation Formula

Friction is the sum of the resistance created by adhesion effects and ploughing effects. Hence, the friction *F* is:

(1) $$F=T+P_{e}=A_{r}\cdot \tau_{b}+S\cdot p_{e}$$

where *T* and *P_e_* are adhesion resistance and ploughing resistance, respectively; *p_e_* is ploughing resistance unit area; *τ_b_* is shear strength at the adhesion point; *S* is ploughing contact area; *A_r_* is adhesion contact area.

## 3. Discretization of Asperities in a Hard–Soft Interface

**Figure 3:**
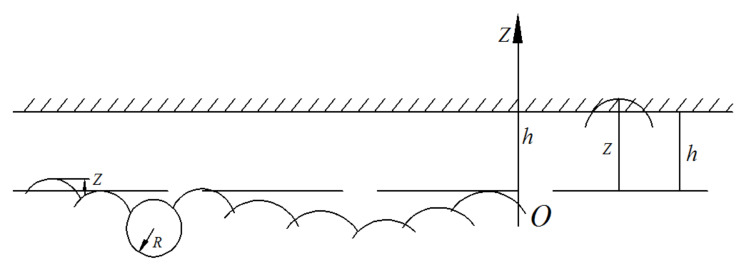
Greenwood and Williamson random surface model.

When considering the hard-soft interface with uniform material properties, it can be assumed that the height of the actual asperities on hard and soft surfaces performs normal distribution function. Therefore, the contacting asperities can be calculated based on the probability. The standard deviation of the asperity height of the hard and soft interface is *σ*_1_ and *σ*_2_, respectively, and *h* is the distance between the center lines of the two surfaces’ contact. The contact between two interface asperities can be transferred into the contact between a smooth rigid surface and a two-dimensional normal distribution surface for which standard deviation of the height distribution is $\sigma =\sqrt{\sigma_{1}^{2}+\sigma_{2}^{2}}$. As shown in Figure 3, the outline of asperities is represented by the Greenwood and Williamson random surface model (GW model) [4] with a radius of curvature *R* and an elastic modulus *E’*. The problem of the actual contact area of the hard-soft interface is transferred into the height distribution problem of the particles which formed asperities.

The adhesion contact occurs in the portion where the asperities height *z > h*:

(2) $$P(z>h)=\int_{h}^{\infty }\Psi \left(z\right)dz$$

If the total number of asperities is *n*, then the contacting asperities number *m* is

(3) $$m=n\int_{h}^{\infty }\Psi \left(z\right)dz$$

The normal deformation of each contact point is *z − h*. According to the contact mechanics, the contact area of a single asperity is obtained and then multiplied by the contacting asperities number *m*, obtaining the actual contact area *A_r_*.

(4) $$A_{r}=m\pi R\left(z-h\right)=n\pi R\int_{h}^{\infty }\left(z-h\right)\Psi \left(z\right)dz$$

Converting the contact interface between two random surfaces into a smooth interface and a random surface lays the foundation for the experiment. Using this result, the experiment can use a smooth surface and a random surface to observe the surface profile, reducing a random variable and reducing the test difficulty. It can be seen from Equation (4) that the contact area is related to the surface profile distribution at the height of the interface (*z − h*).

## 4. White Light Interferometry Test of Clay Soil

**Figure 4:**
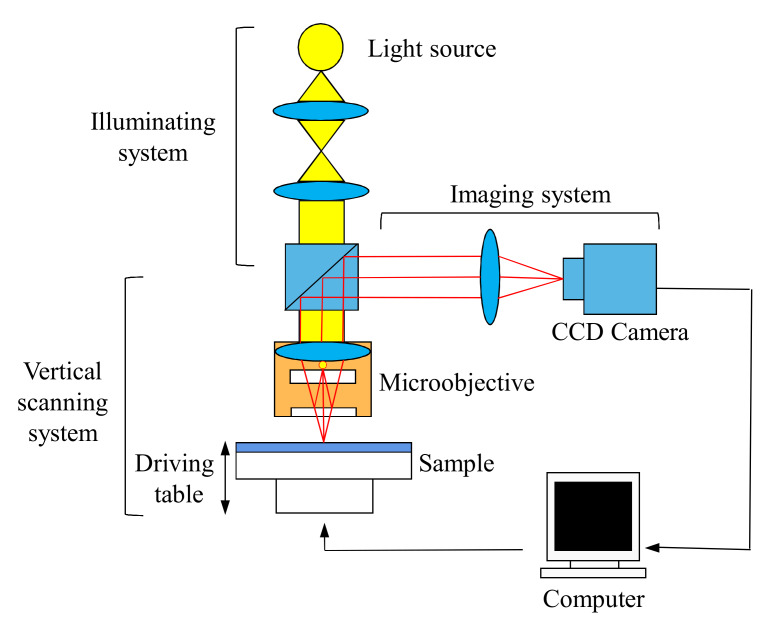
White light interferometry system.

The basic principle of white light interference is to use the same light source to emit two beams of coherent white light at a fixed distance to form interference fringes as the reference optical path. The interference fringes formed on the surface of the measured object due to the difference in profile are used as the detection optical path. By calculating the change of the optical path difference between the reference optical path and the detection light path, the height change of the surface profile is obtained. The white light interferometry system is shown in Figure 4.

The 3D-20 surface profiler from the well-known manufacturer Zeta was used in the test. The measurement accuracy in the XY direction was ≤0.1 μm, and the measurement accuracy in the *Z*-direction was <20 nm. In order to overcome the difficulty of sample handling and eliminate other interference factors, higher compressive stress was adopted in sample preparation.

**Figure 5:**
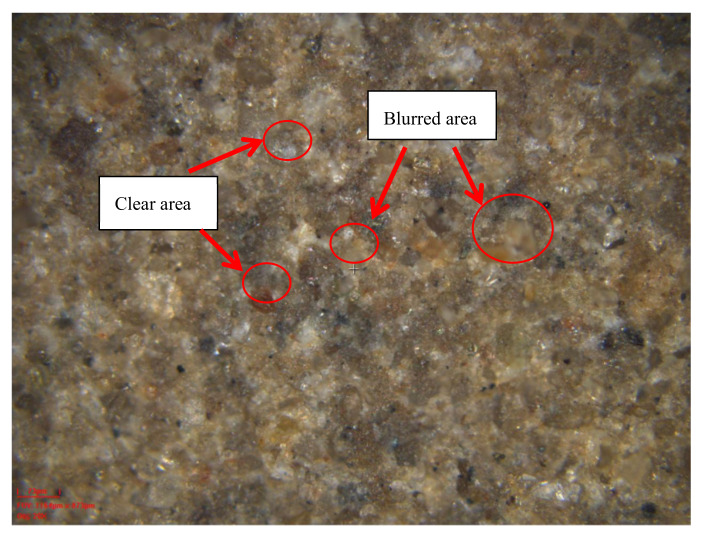
The scanned image at a specific height.

The white light interference instrument needs to emit high-frequency white light at each specific height to scan the entire area. After the specific height scan is completed, the next height scan is performed until all the scans within the set scan height range are completed. Due to the fixed focal length, the image with a specific height matching the focal length is clear, and the images outside the specific height are blurred, as shown in Figure 5.

### 4.1. Sample Preparation

Clay soil used in this experiment was collected from the Jiaozhou Bay of Jiaodong Peninsula, China. The original clay soil possessed a flowing plastic to soft plastic condition with a slight gloss reaction. The specific parameters of the original clay soil are shown in Table 1.

**Figure 6:**
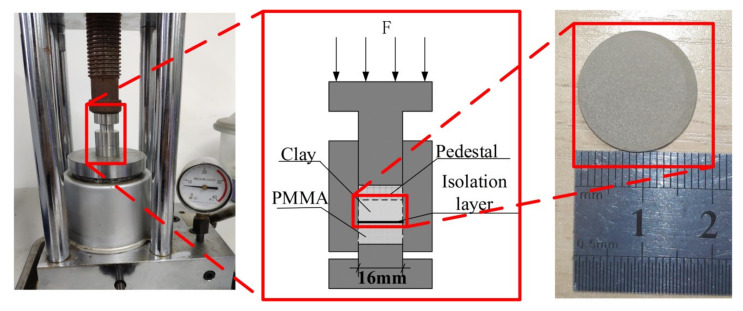
Fabrication of clay soil specimen.

The soil sample after drying and sieving was put into the mold and compressive stress was applied through new polymethyl methacrylate (PMMA) as a smooth and rigid plane. The role of PMMA is the same as that of the top rigid plane shown in Figure 3. The new PMMA used in the test ensured a smooth surface with a diameter of 8.5 mm. In order to make the granular materials have obvious contact, higher compressive stress was set. A total of five samples were made, and the applied normal forces were 2.4 t, 4.8 t, 7.2 t, 9.6 t, and 12 t. The compressive stresses were 106 MPa, 211 MPa, 317 MPa, 423 MPa, and 529 MPa. The whole sample preparation process is shown in Figure 6.

### 4.2. Experimental Measurement

**Figure 7:**
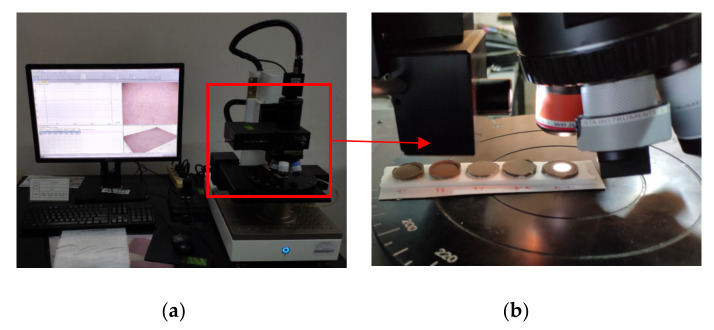
White light interferometry using Zeta 3D surface topography instrument. (**a**) White light interferometer; (**b**) Sample scanning.

Specimens were placed in the center of the operation platform (as shown in Figure 7). A proper horizontal and vertical position of the lens was selected to capture the viewing surface. By adjusting the focal length, the sharpness of the image changes. During the process of adjusting the focal length from far to near and then from near to far, the image changes from blurred to clear and then clear to blurred. The critical point where the image changes from blurred to clear is the starting height of the scan, and the critical point that changes from clear to blurred is the ending height of the scan. The number of scanning steps is set according to the required accuracy and the height difference between the upper and lower limits of the scan. The accuracy of each step in this experiment was about 1 μm.

### 4.3. Particle Size Distribution by Laser Measurement

**Figure 8:**
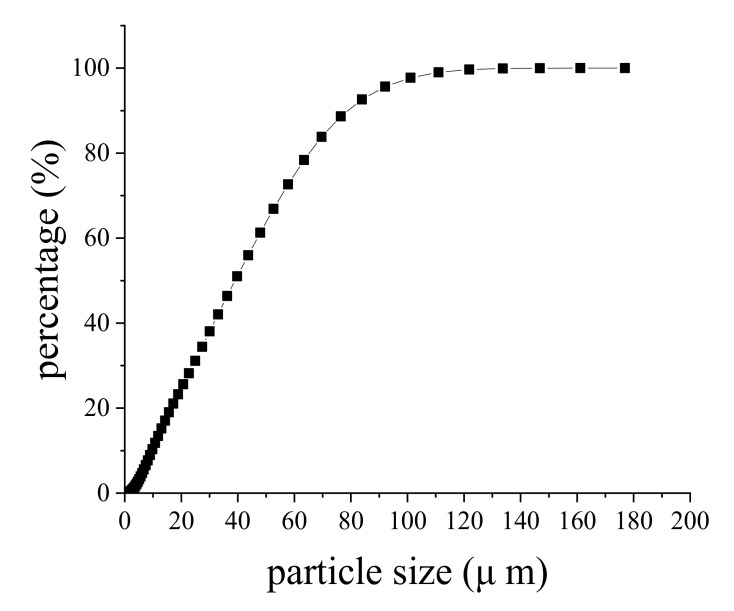
The curve of particle size distribution.

In order to accurately obtain the soil particle distribution, the clay soil after drying and sieving were subjected to laser particle size analysis. The laser particle size analyzer used in the test was the model Mastersizer 3000, which is measured by the dry method. The analysis results are summarized in Table 2 and the curve of particle size distribution is shown in Figure 8.

### 4.4. Profile Data Processing

The profile data were processed as follows:

- A partial section (1500 μm × 1500 μm) in the center of the scanning area (4687 μm × 3516 μm) was selected for analyzing, with a total 45,000 points in this area.
- Horizontal correction was done by the linear fitting of profile data, thereby avoiding the inclination of contour induced from specimen fabrication and placement. The slope was restricted by 2%, which roughly met the experimental requirements.
- Statistics analysis on the point cloud data of *Z*-direction was conducted by taking 5 μm as an interval. A support point height *z*_0_ with the highest distribution frequency was identified regardless of different vertical stress.

## 5. Discussion

### 5.1. Actual Contact Plane Location

**Figure 9:**
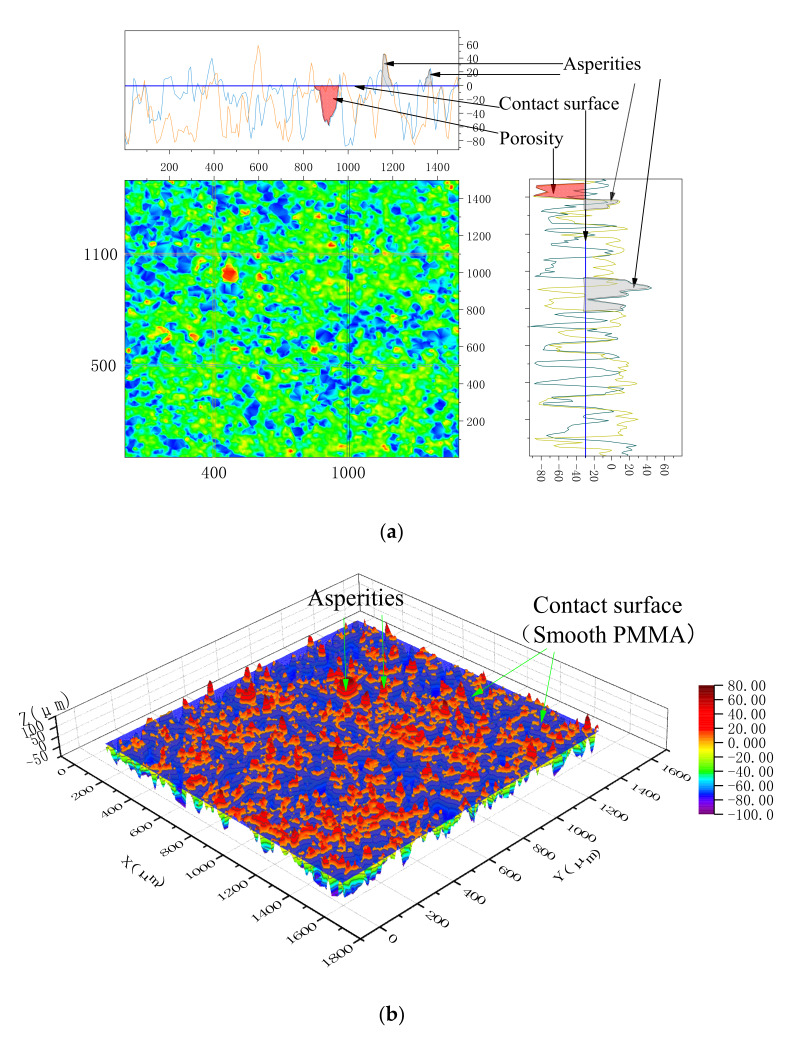
Illustration of the contact plane under compression. (**a**) 2D profile; (**b**) 3D profile.

Loose soil particles were compressed under high stress. With an increase of vertical stress, the growth rate of effective stress between soil particles was lower than that of total stress. In order to bear the additional compressive stress Δ*P*, the height of the outer surface contour decreased and the actual contact area increased Δ*S*. Since the contact surface was pressed by a smooth PMMA, the height of the actual contact plane contour was unified, as illustrated in Figure 9.

There existed a unified profile height for the bearing surface. In other words, it was the point with the highest distribution frequency, defined as the support point height *z*_0_. Particle size distribution was combined in the location of the actual contact surface. The average particle diameters *d*_10_, *d*_25_, *d*_50_, *d*_75_, *d*_90_ were chosen to calculate the cumulative frequency of apparent profile height under various compression stresses (as shown in Table 3).

### 5.2. The Relationship between Actual Contact Area and Overburden Stress

**Figure 10:**
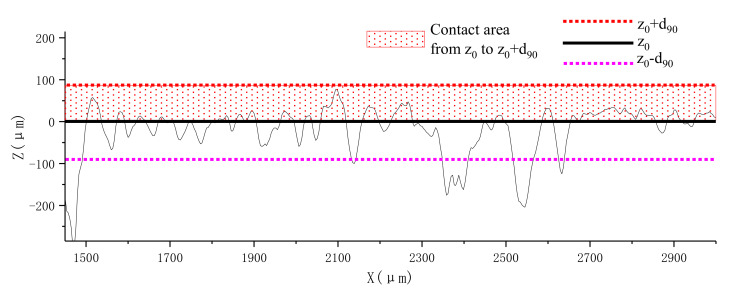
Real contact region located in the 2D profile.

**Figure 11:**
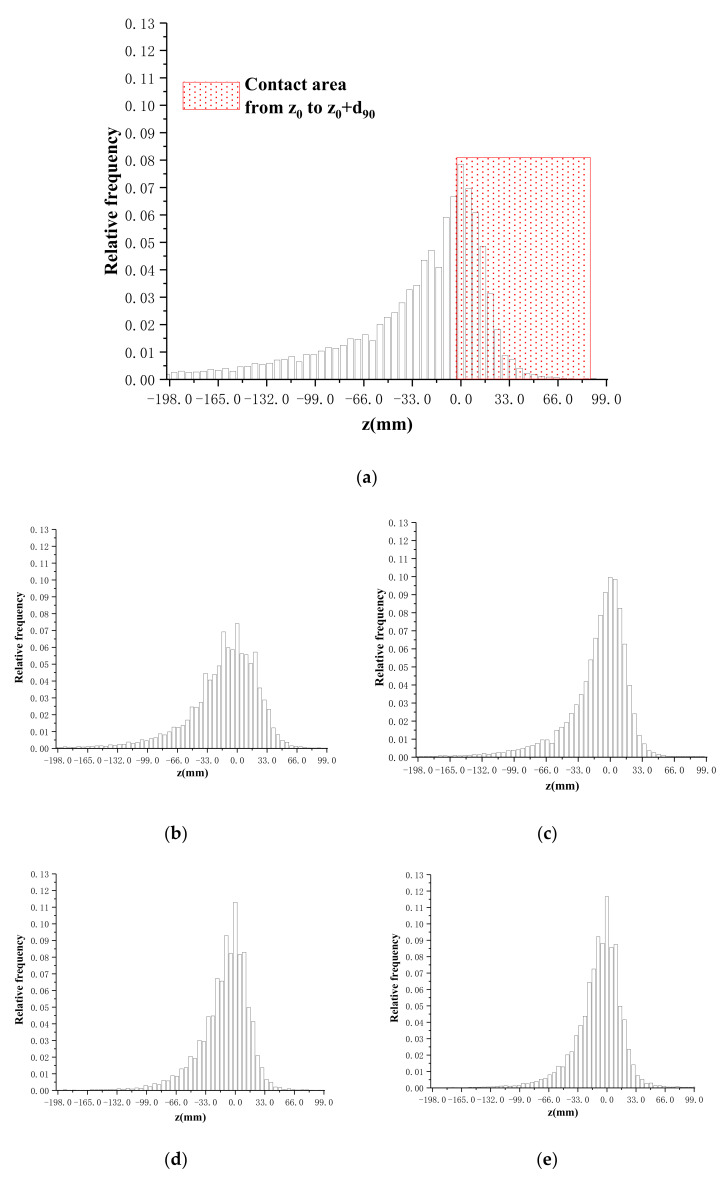
Frequency histogram corresponding to various height in the *Z* direction. (**a**) 106 MPa; (**b**) 211 MPa; (**c**) 317 MPa; (**d**) 423 MPa; (**e**) 529 MPa.

As defined above, the support center *z*_0_ should be the height of the actual bearing surface, hence, there should be no contour above this height theoretically. However, the scanning results indicate that 96% of profile height is distributed in the range from *z*_0_ − *d*_90_ to *z*_0_ + *d*_90_, rather than a simple cross-sectional area with a certain height. This can be explained by the rebound effect of the soil particles after unloading. Hereby, a concept of real contact region (from *z*_0_ to *z*_0_ + *d*_90_) is proposed in this study, where all the particles undertake the external pressure in a cooperative working mechanism. The actual contact area ratio should be the accumulated frequency from *z*_0_ to *z*_0_ + *d*_90_, in which *d*_90_ is the representative value of particle size. The real contact region located in the profile is illustrated in Figure 10, while Figure 11 shows its distribution in the frequency histogram.

**Figure 12:**
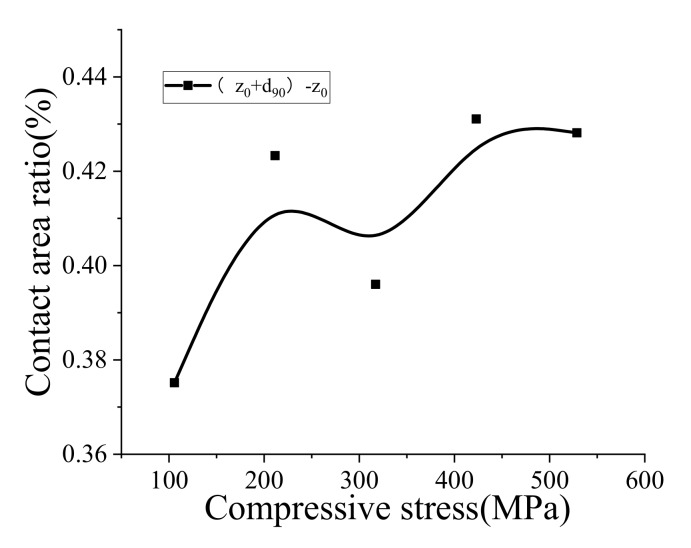
Relationship between the actual contact area ratio and compression stress.

Table 4 shows the accumulated frequency of surface profile near the support center. From Figure 12, it can be seen that the actual contact area ratio is relatively stable, changing from 0.375 to 0.431, performing a slightly increasing trend with the rise of overburden stress.

**Figure 13:**
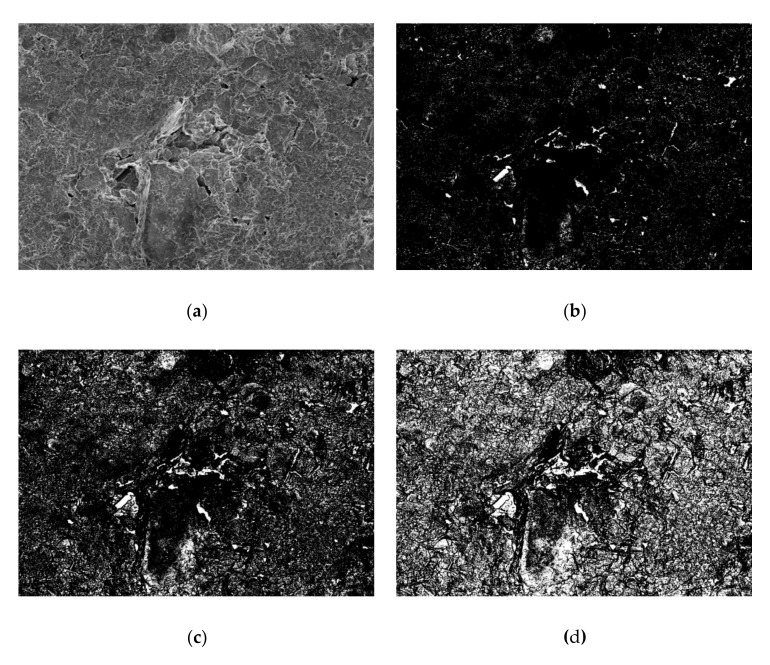
Analysis of SEM image by binarization method for the clay soil compacted under 106 MPa vertical pressure. (**a**) Original SEM image; (**b**) CAR = 98.39% (Gray value = 40); (**c**) CAR = 92.57% (Gray value = 60); (**d**) CAR = 79.56% (Gray value = 80).

For the sake of making a comparison between the contact area ratio (CAR) produced by SEM and white light interferometry, in this study, the binarization method was also used in analyzing the original SEM image of clay soil under 106 MPa compressive pressure. As shown in Figure 13, the arbitrary choice of gray value can bring about a significant variation of the contact area ratio. More specifically, when the gray value changed from 40 to 80, the contact area ratio decreased drastically from 98.39% to 79.56%. Hereby, it is can be proven the contact area ratio calculated directly from the 3D image of white light interferometry is more reliable than that produced from the binarization of the SEM image.

### 5.3. Apparent Porosity and Compressive Stress

**Figure 14:**
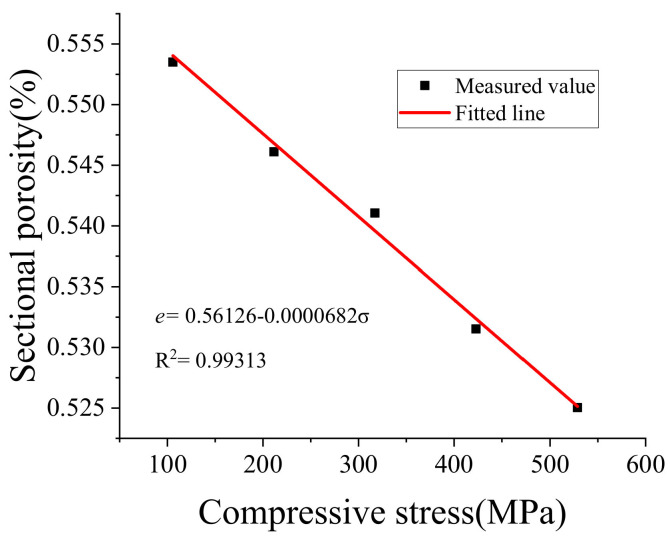
The relationship between apparent porosity and compressive stress.

The pores near the support center change as the stress increases. According to the surface profile data of each point obtained by the white light interference, Equation (5) calculates the difference between the *z*_0_ + *d*_90_ height and the height of each point, and integrates over the entire range to calculate the porosity. Equation (5) is used to calculate the volume integration in the range of *d*_90_, above and below the support center *z*_0_. The schematic diagram of apparent porosity calculation is illustrated in Figure 14.

(5) $$e=\frac{\int_{z_{0}-d_{90}}^{z_{0}+d_{90}}\left[\left(z_{0}+d_{90}\right)-z\right]dz\int \int dxdy}{{\int \int \int }_{z_{0}-d_{90}}^{z_{0}+d_{90}}dzdxdy}$$

where *e* is the apparent porosity.

The relationship between apparent porosity and compressive stress is illustrated in Figure 13. It shows that the apparent porosity decreased linearly with increasing stress, but at a slower rate as observed through the amplifying relationship.

**Figure 15:**
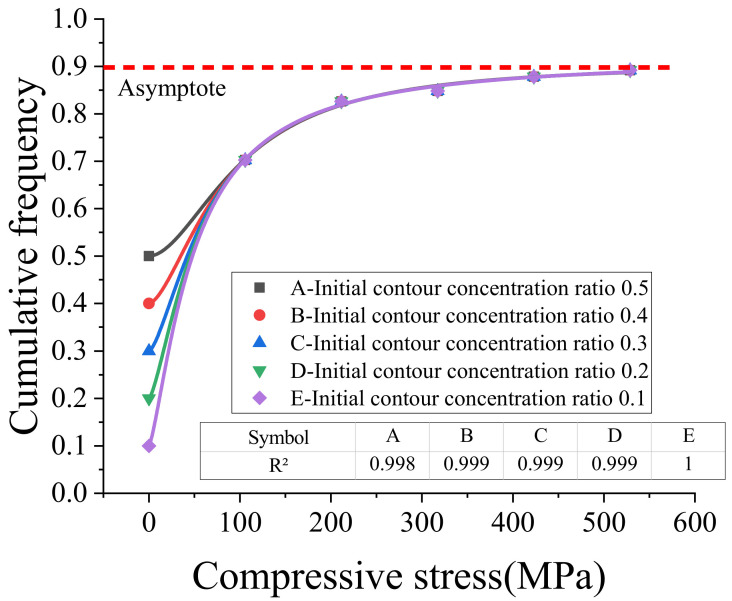
Regression curve of contour distribution near the support center.

Table 4 also shows that with an increase of compressive stress, the cumulative frequency of apparent profile height (*z*_0_ − *d*_90_ − *z*_0_ + *d*_90_) had a concentrated tendency with a limited value, which resulted from the stabilization of porosity. A logistic function was used to derive this limit asymptotic value under the different initial accumulated frequency of apparent profile; the limit asymptotic value was about 0.9. The fitted curve is shown in Figure 15.

**Figure 16:**
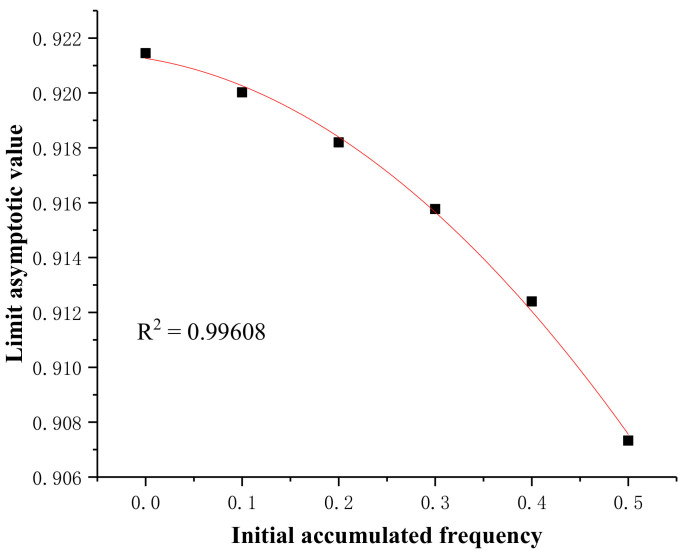
Relationship between the initial accumulated frequency of apparent profile and its corresponding limit asymptotic value.

The data in Table 5 show that, as the initial accumulated frequency of apparent profile (zero stress or natural accumulation state) increased near the support center, the limit asymptotic value was reduced and maintained at a high level of 0.907–0.921. The fitted curve and corresponding fitted equation are illustrated in Figure 16 and Equation (6), respectively. This observation provides an experimental basis for the PFC (Particle Flow Code) simulation.

(6) $$y=0.92126-0.00561x-0.04357x^{2}$$

## 6. Conclusions

An innovative mesoscopic study on the surface profile of clay soil was conducted in this study by white light interference. The concept of real contact region (from *z*_0_ to *z*_0_ + *d*_90_) was proposed when considering the rebound of soil particles. It was proven that the proposed new method can accurately reveal the real contact area ratio (CAR), which is indispensable in the modern tribology theory of adhesion-ploughing. The stresses used in the present experiments were all over 100 MPa, and stresses of this magnitude mostly appear in the contact between the tips of the soil particles, and rarely occur in the large-area contact in engineering. The particle contact under stress commonly used in engineering still needs further study. Based on the experiments presented, the following conclusions can be drawn:

1. Under high compressive stress, the real contact area ideally should be the total area of all soil particles in the real contact region (from *z*_0_ to *z*_0_ + *d*_90_), when considering the rebound of the soil particles after unloading, rather than a simple cross-sectional area with a certain height. Hence, compared with the traditional SEM, white light interferometry has an obvious advantage to reveal the real contact area, which would be quite useful in the mesoscopic research of soil particles friction.
2. The actual contact area ratio of clay soil is in the range of 0.375–0.431, and the apparent porosity is approximately 0.525–0.554, demonstrating a linear increase with the rise of overburden pressure.
3. With an increase of compressive stress, the cumulative frequency of apparent profile height (from *z*_0_ − *d*_90_ to *z*_0_ + *d*_90_) has a concentrated tendency with limited value, and a logistic function is used to derive this limit asymptotic value.
4. A regression formula for estimating the limit asymptotic value under various initial values was established for clay soil, which provides an experimental basis for the DEM (Discrete Element Method) simulation.

## Figures and Tables

**Table 1:** Physical and mechanical parameters of clay soil sample.

*d_s_* (g/cm^3^)	*Γ* (kN/cm^3^)	*W* (%)	*w*_L_ (%)	*w*_p_ (%)	*I*_p_ (%)	Cohesion Force (kPa)	*IFA* (o)	*E_s1-2_* (MPa)
2.73	18.0	34.8	34.8	21.2	13.5	14.4	8.6	3.3

**Table 2:** Particle size representative value.

Level	*d*_10_	*d*_25_	*d*_50_	*d*_75_	*d*_90_
Particle size (μm)	10.55	22.21	42.82	65.95	86.69

*d*_10_ represents the particle size corresponding to 10% of the volume fraction.

**Table 3:** Cumulative frequency of contact surfaces of each height under high stress of clay soil.

Pressure (MPa)	Cumulative Frequency (CF)										
	*z*_0_ − *d*_90_	*z*_0_ − *d*_75_	*z*_0_ − *d*_50_	*z*_0_ − *d*_25_	*z*_0_ − *d*_10_	*z*_0_	*z*_0_ + *d*_10_	*z*_0_ + *d*_25_	*z*_0_ + *d*_50_	*z*_0_ + *d*_75_	*z*_0_ + *d*_90_
106	0.280	0.332	0.414	0.496	0.556	0.608	0.662	0.7540	0.881	0.963	0.983
211	0.139	0.193	0.296	0.402	0.477	0.542	0.591	0.671	0.807	0.916	0.965
317	0.138	0.191	0.284	0.413	0.416	0.590	0.657	0.785	0.916	0.974	0.986
423	0.110	0.163	0.258	0.387	0.495	0.557	0.643	0.745	0.894	0.970	0.988
529	0.088	0.136	0.234	0.371	0.479	0.552	0.652	0.754	0.891	0.964	0.980

**Table 4:** Cumulative frequency of surface profile near the support center.

Pressure (MPa)	Cumulative Frequency (CF)		
	(*z*_0_ + *d*_90_) − *z*_0_	*z*_0_ − (*z*_0_ − *d*_90_)	(*z*_0_ + *d*_90_) − (*z*_0_ − *d*_90_)
106	0.375	0.328	0.703
211	0.423	0.403	0.826
317	0.396	0.452	0.848
423	0.431	0.447	0.878
529	0.428	0.464	0.892

**Table 5:** Relationship between initial contour cumulative frequency and limit contour CF.

Initial Contour (*z*_0_ + *d*_90_) − (*z*_0_ − *d*_90_) CF	R^2^	Limit Profile (*z*_0_ + *d*_90_) − (*z*_0_ − *d*_90_) CF
0.1	1	0.920 ± 0.026
0.2	0.999	0.918 ± 0.025
0.3	0.999	0.916 ± 0.024
0.4	0.999	0.912 ± 0.022
0.5	0.998	0.907 ± 0.020
